# Calpain1 inhibition enhances autophagy-lysosomal pathway and ameliorates tubulointerstitial fibrosis in Nephronophthisis

**DOI:** 10.1186/s10020-025-01231-4

**Published:** 2025-05-03

**Authors:** Dantong Li, Jinglan Zhang, Xinyu Su, Yichen Yang, Jiayong Lai, Xiaoya Wei, Huamu Chen, Yaqing Liu, Haiyan Wang, Liangzhong Sun

**Affiliations:** 1 Department of Pediatrics, Nanfang Hospital, Southern Medical University, Guangzhou, 510515 China; 2 Department of Nephrology, Guangdong Provincial People’s Hospital (Guangdong Academy of Medical Sciences), Southern Medical University, Guangzhou, 510080 China; 3 Department of Pediatrics, Guangzhou First People’s Hospital, School of Medicine, South China University of Technology, Guangzhou, 510080 China; 4 Department of Pediatrics, First Affiliated Hospital of Gannan Medical University, Gannan Medical University, Ganzhou, 341000 China; 5 Department of Pediatrics, Sun Yat-Sen Memorial Hospital, Sun Yat-Sen University, Guangzhou, 510120 China

**Keywords:** Calpain1, Renal ciliopathies, Renal tubulointerstitial fibrosis, Inherited kidney disease, Nephronophthisis

## Abstract

**Background:**

Nephronophthisis (NPH) is classified under the category of renal ciliopathies and is the most common genetic disease leading to renal failure in children. Early-onset and progressive renal tubulointerstitial fibrosis represents one of the most significant features, culminating in renal insufficiency. However, the molecular mechanism of tubulointerstitial fibrosis remains unclear. Previously, we constructed an NPH mouse model via CRISPR-Cas9. This mouse model demonstrated typical features of tubulointerstitial fibrosis. In this study, we aimed to explore the pathogenesis of tubulointerstitial fibrosis in NPH and identify early intervention targets in both the NPH models and patients.

**Methods:**

In this study, transcriptome changes in mouse kidneys were analyzed through RNA sequencing to explore the molecular mechanisms of renal tubulointerstitial fibrosis in NPH. We found an increased abundance of calpain1 in both the NPH models and patients. Pathway enrichment analysis indicated autophagy-lysosomal pathway was altered in the NPH models. Western blot, immunofluorescence or immunohistochemical staining were used to verify the expression of calpain1. We also detected autophagy activities in NPH models by lysotracker staining and transmission electron microscopy (TEM). Epithelial or mesenchymal-specific markers and Masson’s trichrome staining were used to detect the status of tubulointerstitial fibrosis. Furthermore, NPH models were treated with a calpain1 inhibitor to explore the role of calpain1 in autophagy-lysosomal pathway and tubulointerstitial fibrosis.

**Results:**

The increased abundance of calpain1 impaired the autophagy-lysosomal pathway and induced tubulointerstitial fibrosis by promoting epithelial-to-mesenchymal transition. On the other hand, calpain1 inhibition could enhance the autophagy-lysosomal pathway and ameliorate the phenotypes of tubulointerstitial fibrosis in NPH models.

**Conclusions:**

Calpain1-mediated autophagy-lysosomal pathway disorder may be an important cause of tubulointerstitial fibrosis in NPH. Calpain1 may have therapeutic implications for renal tubulointerstitial fibrosis.

**Supplementary Information:**

The online version contains supplementary material available at 10.1186/s10020-025-01231-4.

## Background

Renal ciliopathies usually lead to progressive chronic kidney disease and end-stage kidney disease (ESRD). Nephronophthisis is an autosomal-recessive ciliopathy and represents one of the most frequent inherited causes of kidney failure in childhood. NPH patients progress to ESRD at an early age Braun and Hildebrandt 2017; Kagan et al. 2017. Owing to the high cost of dialysis and the shortage of kidney transplantations, NPH brings formidable challenges to patients, their families and healthcare providers. Therefore, it is significant to explore the pathogenesis of NPH and identify early intervention targets.

There are more than 26 identified pathogenic genes of NPH. *NPHP1*is the most prevalent gene and accounts for the majority of cases. Early-onset and progressive renal tubulointerstitial fibrosis represents one of the most significant phenotypes of NPH and plays an essential role in disease progression Petzold et al. 2023. Thus, figuring out the molecular basis of tubulointerstitial fibrosis might be conducive to ameliorating the progression of chronic kidney disease and decreasing the risk of end-stage kidney disease.

Previously, we successfully constructed an *Nphp1*knockout mouse model via CRISPR-Cas9 Li et al. 2021. To identify transcripts of interest, we applied RNA-seq to kidneys from the NPH mouse model. Transcriptomic data revealed prominent upregulation of CAPN in the kidneys of NPH model mice. CAPN encodes calpains which are calcium-activated proteases that exist in the cytosol. There are more than 15 homologous members in the calpain family. They distinctively expressed in different tissues and organs of different species Macqueen and Wilcox 2014. Calpains have been implicated in the pathogenesis of many human diseases, such as blood clotting disorders Azam 2120, Alzheimer’s disease Wang et al. 2022; Guo et al. 2022, limb-girdle muscular dystrophy Difranco et al. 2016, irreversible blindness Mahajan et al. 2012 and potential involvement in cancer Leloup and Wells 2011, as well as kidney diseases Chatterjee et al. 2001; Hanouna et al. 2017. In autosomal dominant polycystic kidney disease (ADPKD), calpains cause diminished lysosomal acidification and play a role in disease progression Peintner et al. 2021.

The mechanisms by which calpains mediate different biological functions are not fully understood. Calpains play a particular role in autophagy Russo et al. 2011, which has been associated with kidney injury in various disease states Zhang et al. 2022; Jiang et al. 2010. The upregulation of calpains can inhibit autolysosomes Nowak and Edelstein 2020, whereas the inhibition of autolysosomes can lead to tubulointerstitial fibrosis in kidney diseases Ding et al. 2014. In this study, we verified the upregulation of calpains in NPH patients and models. When calpain1 was upregulated, the integrity of autolysosomes was destroyed, leading to the interruption of autophagy in*NPHP1*-targeted human kidney-2 (HK2) cell line and mouse kidneys. Furthermore, tubulointerstitial fibrosis was promoted when autophagy was interrupted. However, the autophagy-lysosomal pathway could be enhanced and tubulointerstitial fibrosis could be ameliorated by calpain1 inhibition. Our study aimed to clarify whether calpain1 may be a new target for early-stage NPH diagnosis and therapy.

## Methods

### HK2 cell culture and treatment

The HK2 cell line was purchased from the Public Medical Laboratory Center of Nanfang Hospital, Southern Medical University. The cells were cultured with DMEM (ThermoFisher, Massachusetts, USA) supplemented with 10% fetal bovine serum (ThermoFisher, Massachusetts, USA) and 1% antibiotics (ThermoFisher, Massachusetts, USA), in a humidified incubator with 5% CO2 at 37 °C. High-titer lentiviral shRNA vectors were prepared by Shanghai Jikai Gene Chemical Technology Co. (Shanghai, China) to generate *NPHP1* knockdown HK2 cells, and medium containing puromycin (2 µg/ml) was used to establish stable knockdown(sh*NPHP1*) HK2 cell lines. The cells were treated with the calpain1 inhibitor, calpeptin at a concentration of 25 μM (Selleckchem, Shanghai, China) for 48 h. To inhibit autophagic influx, cells were incubated with medium supplemented with Bafilomycin A1 (200 nM) (Selleckchem, Shanghai, China) for 24 h.

### Isolation and cultivation of renal epithelial cells from urine

Renal epithelial cells were isolated from 100–200 mL of urine from healthy donors and NPH patients by centrifugation at 400 × g for 10 min. After that, the cells were washed with 50 μg/mL gentamicin and 250 μg/mL amphotericin B (ThermoFisher, Massachusetts, USA) containing Dulbecco’s PBS (ThermoFisher, Massachusetts, USA) and centrifuged at 200 × g for 10 min. Then, the cells were suspended with primary medium and plated in 12-well plate. The primary medium consists of high-glucose DMEM/F12, 10% fetal bovine serum (ThermoFisher, Massachusetts, USA), 10% UrinEasy isolation culture supplement (Cellapy, Beijing, China), 50 μg/mL gentamicin and 250 μg/mL amphotericin B. For the next 3 d, 1 mL of primary medium was added each day. The obtained cells were then cultivated in UrinEasy proliferation medium (Cellapy, Beijing, China). The cells were cultivated at 37 °C with 5% CO_2_, and the medium was changed every 2–3 days.

### Animal studies

*Nphp1*^−/−^C57BL/6 J mouse models were constructed as described in our previous research Li et al. 2021. C57BL/6 J mice were housed at the animal facility located in Nanfang Hospital, Southern Medical University on a 12 h/12 h light/dark cycle at 21 °C and 50%–55% humidity under specific pathogen-free (SPF) conditions with food and water available ad libitum. At the age of 8 weeks, male or female mice were randomly numbered and grouped via a random number table method. Wild-type and*Nphp1* knockout mice were randomly divided into 3 groups. They were treated as follows: (i) control group: no treatment; (ii) calpeptin group: intraperitoneal injections with calpeptin (2 mg/kg) every 4 d for 16 weeks; (iii) Bafilomycin A1 group: intraperitoneal injections with Bafilomycin A1 (1 mg/kg) every 4 d for 16 weeks. Individual weights were taken on all mice once a week until sacrifice. 24-week-old mice were anesthetized with 3% pentobarbital sodium and sacrificed.

### RNA-Seq

Total RNA was isolated from whole kidney tissues of *Nphp1*^+/+^ and *Nphp1*^−/−^ C57BL/6 J mice (*n* = 3 per group) with TRIzol Reagent (ThermoFisher, Massachusetts, USA). Complementary DNA libraries were constructed via an Illumina TruSeq RNA Sample Prep kit according to the manufacturer’s protocol. High-throughput RNA sequencing (RNA-seq) was performed via an Illumina HiSeq 4000 (Illumina, California, USA) at CapitalBio Corporation (Beijing, China). The read alignment was conducted via TopHat 2.0.13, and relative transcript abundances and differentially expressed genes (DEGs) were determined via the DESeq R package (1.3.0). A *p* value < 0.05 and a fold change cutoff as a |log_2_ ratio|> 1 were used for the detection of DEGs via RNA-seq analysis. The analyzed results were visualized via the ImageGP tool (http://www.ehbio.com/ImageGP/).

### RNA extraction and RT-PCR

Total RNA was isolated with TRIzol reagent (ThermoFisher, Massachusetts, USA) and then reverse transcribed into cDNA according to the manufacturer's procedures (Accurate Biology, Changsha, China). Relative mRNA expression was analyzed via SYBR Green Pro Taq HS (Accurate Biology, Changsha, China) with an Applied Biosystems QuantStudio 6 Flex Real-Time PCR System (ThermoFisher, Massachusetts, USA). β-actin (Accurate Biology, Changsha, China) was used for normalization. The expression of specific genes was quantified via the ΔΔCt method. The primers used were as follows: mouse *Capn11* (5'‐AAACTGCTGTACCGTGTGGTG‐3′ and 5'‐GTTCAACCAGTGCCCAAACTG‐3′), human *CAPN1* (5'‐GGGTCCCAATTCCTCCAAGA‐3′ and 5'‐CTGGAAATGGAAGATGCCGG‐3′), mouse *Capn1* (5'‐CCGTGGACTTTGACAACTTTG‐3′ and 5'‐CCCCACTTCAGGCAAACATAG‐3′), S100 A4 (5′-CAGAACTAAAGGAGCTGCTGACC-3′ and 5′-CTTGGAAGTCCACCTCGTTGTC-3′), ACTA2 (5′-CTATGCCTCTGGACGCACAACT-3′ and 5′-CAGATCCAGACGCATGATGGCA-3′).

### Protein extraction and Western blot

Proteins were extracted from the tissue and cell samples and used for western blot analysis. Cells and tissues were lysed with Radio Immunoprecipitation Assay (RIPA) lysis buffer (Beyotime, Jiangsu, China) containing a protease inhibitor cocktail and a phosphatase inhibitor cocktail. The amount and quality of proteins were determined via a bicinchoninic acid (BCA) assay (ThermoFisher, Massachusetts, USA) and a Synergy H1 Microplate Reader (BioTek, Washington, USA). The quantified protein samples were separated via electrophoresis on an SDS–polyacrylamide gel before being transferred onto polyvinylidene fluoride (PVDF, Sigma Aldrich, California, USA) membranes. The membranes were blocked in 5% skim milk (Solarbio, Beijing, China) for 1 h at room temperature. They were then incubated with a specific antibody, followed by incubation with a secondary antibody conjugated to horseradish peroxidase. The following primary antibodies were used: anti-calpain1 (CAPN1, Proteintech, Chicago, USA), anti-glyceraldehyde 3-phosphate dehydrogenase (GAPDH, Proteintech, Chicago, USA), anti-nephrocystin1 (NPHP1, Sigma Aldrich, California, USA), anti-lysosomal-associated membrane protein 1 (LAMP1, Affinity Biosciences, Ohio, USA), anti-lysosomal-associated membrane protein 2 (LAMP2, Affinity Biosciences, Ohio, USA), anti-sequestosome 1 (P62, Proteintech, Chicago, USA), anti- light chain 3 (LC3, Cell Signaling Technology, Massachusetts, USA), anti-fibroblast-specific protein 1 (FSP-1, Proteintech, Chicago, USA), anti-alpha smooth muscle actin (α-SMA, Cell Signaling Technology, Massachusetts, USA), anti-β-catenin (Abmart, Shanghai, China), anti-E-cadherin (BD Biosciences, San Francisco, USA) and anti-Collagen-I (Abcam, Cambridgeshire, USA).

### Histology and immunohistochemical staining

Kidneys were collected and fixed with 4% paraformaldehyde at 4 °C overnight. Then they were treated with an alcohol series and xylene before being embedded in low-melting paraffin. Sections (4 μm) were deparaffinized with xylene and rehydrated in a graded alcohol series. Some sections were subjected to Masson’s trichrome staining (Solarbio, Beijing, China) for morphological assessment. Some sections were subjected to immunohistochemistry (IHC). Briefly, antigen retrieval was applied to perform immunohistochemistry by heating sections immersed in Ethylenediaminetetraacetic acid (EDTA) solution (pH 9.0) in a pressure cooker for 10 min. The sections were then blocked with 2% bovine serum albumin (BSA) at room temperature for 60 min and incubated with a specific primary antibody at 4 °C overnight. Then, the sections were incubated with a goat anti-rabbit secondary antibody labeled with biotin for 30 min at 37 °C. The sections were stained with 3,3-diaminobenzidine (DAB) before counterstaining with hematoxylin. Brownish yellow granules in the cytoplasm were interpreted as positive regions. Images were taken with a microscope (OLYMPUS N21773, Japan) or the Slide Scanning System SQS-40R (Teksqray, China).

For the quantification of Masson’s trichrome staining, Color Threshold tool was used to separately select the blue (collagen fibers) and red regions in ImageJ software (Ver1.8.0, NIH, Bethesda, Maryland, USA). The collagen fiber area percentage is calculated as: Collagen area percentage = (Number of blue region pixels/Total tissue area pixels) × 100%. The relative collagen content was calculated using six consecutively selected fields of the renal cortex and medulla (Scale bar = 100 μm) in the renal sections. The collagen area percentage in *Nphp1*^+/+^ group was normalized as the reference standard.

### Immunofluorescence

Tissue (4 μm) or cell samples on glass slides were permeabilized for 15 min with 0.1% Triton X-100 in phosphate-buffered saline (PBS). The samples were subsequently blocked with 10% goat serum for 1 h before they were incubated with primary antibodies overnight at 4 ℃. After that, the sections were incubated with Alexa Fluor® 594-conjugated secondary antibodies. For lysosome detection, LysoTracker™ Red DND-99 (ThermoFisher, Massachusetts, USA) was used. The samples were counterstained with 4′,6-diamidino-2-phenylindole (DAPI, Servicebio, Wuhan, China) for 15 min. The slides were photographed via a microscope (OLYMPUS N21773, Japan) and ImageJ software (Ver1.8.0, NIH, Bethesda, Maryland, USA) was used for analysis.

### Transmission electron microscopy (TEM)

The ultrastructure and autophagy status of HK2 and mouse kidney tissue samples were observed via transmission electron microscopy (TEM, Hitachi H-7500, Japan). Briefly, the samples were treated with 2.5% glutaraldehyde (Nacalai, Kyoto, Japan) in phosphate-buffered saline overnight at 4 °C. The samples were postfixed in OsO4 and then embedded in epoxy resin. Ultrathin 50–100 nm sections of the samples were made with an ultramicrotome and a diamond knife, and then the samples were examined and photographed via TEM. The morphological criteria of the autophagosome and autolysosome were set as follows in accordance with the current consensus: the autophagosome is a double-membrane structure containing undigested contents, and the autolysosome is a single-layer membrane structure formed by the fusion of the autophagosome and lysosome.

### Statistical analysis

All the statistical analyses were performed with GraphPad Prism version 9.0.3. The data were normally distributed, and one-way analysis of variance (ANOVA) followed by the Tukey test was used for comparisons among multiple groups. The data are presented as the mean ± SEM. *p* < 0.05 was considered to indicate a statistically significant difference.

## Results

### The upregulation of calpains in NPH

**Fig. 1:**
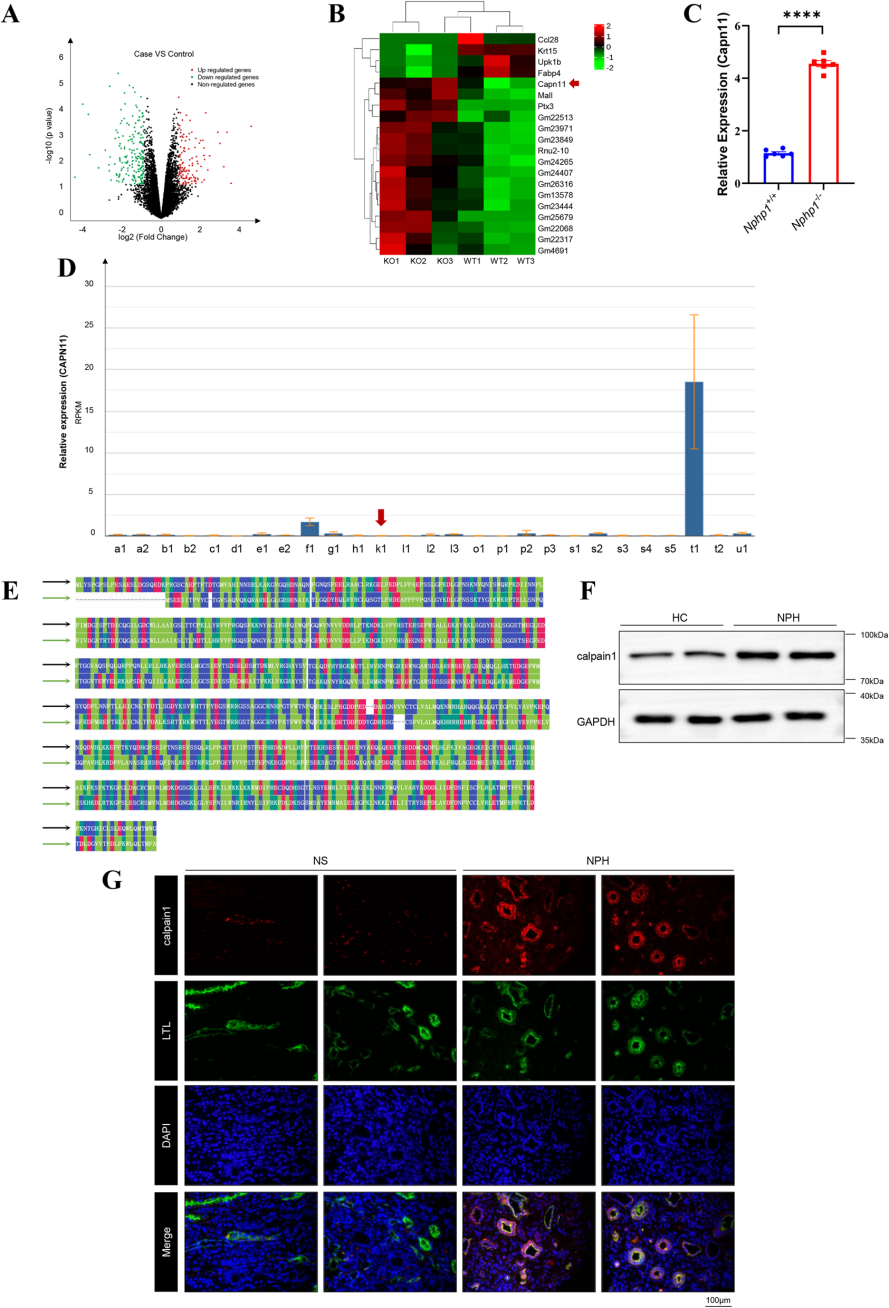
The upregulation of calpains in NPH. **A** Volcano plot of differentially expressed genes (DEGs) in the kidneys of wild-type and *Nphp1* knockout mice (*n* = 3). **B** Heatmap of the top 20 DEGs in wild-type and *Nphp1* knockout mouse kidneys (*n* = 3). **C** RT-qPCR analysis on expression of *Capn11* in in the kidneys of wild-type and *Nphp1* knockout mice (*n* = 6). β-actin was used as an internal control. **D** RNA-seq was performed on tissue samples from 95 human individuals representing 27 different tissues to determine tissue-specificity of *CAPN11*. RPKM, reads per kilobase per million reads placed. a1, adrenal; a2, appendix; b1, bone marrow; b2, brain; c1, colon; d1, duodenum; e1, endometrium; e2, esophagus; f1, fat; g1, gall bladder; h1, heart; k1, kidney; l1, liver; l2, lung; l3, lymph node; o1, ovary; p1, pancreas; p2, placenta; p3, prostate; s1, salivary gland; s2, skin; s3, small intestine; s4, spleen; s5, stomach; t1, testis; t2, thyroid; and u1, urinary bladder. The red arrow represents the expression of calpain11 in the kidney. **E** Multiple sequence alignment (MSA) results for human calpain11 and human calpain1. The black and green arrows represent the amino acid sequences of human calpain11 and human calpain1, respectively. **F** Western blot was used to detect the expression of calpain1 in urine-derived epithelial cells isolated from NPH patients or healthy controls. Samples in the first and second lanes were extracted from urine on two different days but from the same person. Samples in the third and fourth lanes were extracted from urine on two different days but from the same NPH patient. HC, healthy controls; NPH, NPH patients. **G** Immunofluorescence staining of calpain1 (red) and Lotus tetragonolobus lectin (LTL, green) in kidney sections from a patient diagnosed with nephrotic syndrome (NS) and an NPH patient indicating the distribution of calpain1 in proximal tubular epithelial cells. Scale bar = 100 μm

RNA sequencing analysis was used to investigate the gene-expression profile of the NPH mouse model. A *p* value < 0.05 and a fold change cutoff of a |log_2_ ratio|> 1 were used for detection of differentially expressed genes (DEGs). A total of 364 genes were identified (Fig. 1A), and *Capn11* was among the top 20 DEGs in the kidneys of the NPH model mice (Fig. 1B, Table S1). RT-qPCR analysis validated the elevated expression level of *Capn11* in the renal tissue of NPH models (Fig. 1C). Owing to the essential role of calpains in multiple kidney diseases, a possible link between CAPN and the NPH might exist. However, renal expression of *CAPN11* in humans is relatively low (Fig. 1D) according to the Human Protein Atlas (HPA) Fagerberg et al. 2014, a project in which RNA-seq was performed on tissue samples from 95 human individuals representing 27 different tissues to determine the tissue-specificity of all protein-coding genes. The species difference between rodents and humans might explain this discrepancy. Since*CAPN11* has the greatest sequence homology (54.3%) to *CAPN1*catalytic subunit in humans Dear et al. 1999 (Fig.1E, Table S2), a possible link between *CAPN1* and NPH patients might exist. An increase in calpain1 was detected in the renal tubular epithelial cells (Fig. 1F), which were urine-derived epithelial cells from an NPH patient. Moreover, the expression of calpain1 was distributed mainly in proximal tubular epithelial cells (Fig. 1G). This patient demonstrated a homozygous deletion that encompasses the *NPHP1* gene at 2q13 (Fig. S1 A), whereas his parents demonstrated one heterozygous deletion in *NPHP1* (Fig. S1B, C).

**Fig. 2:**
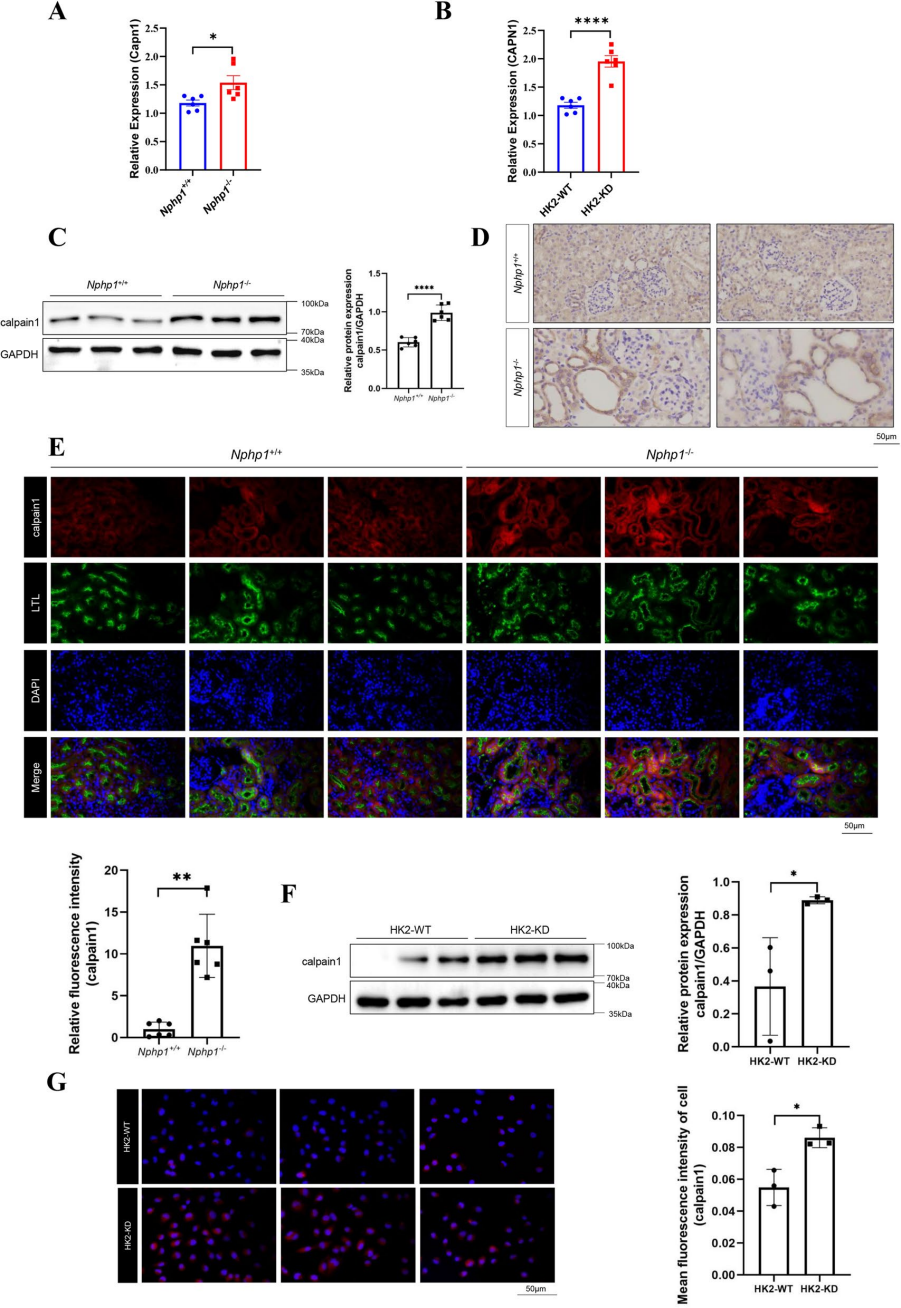
The upregulation of calpain1 in NPH model. **A** RT-qPCR analysis on expression of *Capn1* in in the kidneys of wild-type and *Nphp1* knockout mice (*n* = 6). β-actin was used as an internal control. **B** RT-qPCR analysis on expression of *CAPN1* in in the kidneys of wild-type and *NPHP1* knockdown HK2 cells (*n* = 6). β-actin was used as an internal control. **C** Calpain1 expression in the kidneys of wild-type and *Nphp1* knockout mice was detected via western blot. The result of the quantitative analysis was displayed as bar graphs. *n* = 6 in each group. **D** Calpain1 expression in the kidneys of wild-type and *Nphp1* knockout mice was detected via immunohistochemical staining. Scale bar = 50 μm. **E** Immunofluorescence staining of calpain1 (red) and LTL (green) in the kidneys of wild-type and *Nphp1* knockout mice revealed the distribution of calpain1 in proximal tubular epithelial cells. Scale bar = 50 μm. The results of the quantitative analysis of calpain1 staining in mouse kidneys were evaluated. *n* = 6 in each group. **F** Calpain1 expression in wild-type and *NPHP1* knockdown HK2 cells was detected by western blot. The result of the quantitative analysis was displayed as bar graphs. *n* = 3 in each group. **G** Calpain1 was detected by immunofluorescence in wild-type and *NPHP1* knockdown HK2 cells. The results of the quantitative analysis of calpain1 staining in HK2 cells was evaluated. *n* = 3 in each group, Scale bar = 50 μm. ^*^ P < 0.05; ^**^ P < 0.01; ^***^ P < 0.001; ^****^ P < 0.0001

The upregulation of calpain1 was validated by RT-qPCR (Fig. 2A) western blot (Fig. 2C) and immunohistochemical staining (Fig. 2D) in the kidneys of NPH model mice. Moreover, the expression of calpain1 was distributed mainly in proximal tubular epithelial cells (Fig. 2E, S2 A). HK2 cells are a tubular cell line derived from a normal human adult male kidney. To reduce the resulting bias that may be caused by species differences, we carried out in vitro experiments in HK2 cells. RT-qPCR (Fig. S3 A) and western blot (Fig. S3B) results confirmed the stable knockdown efficacy of *Nphph1* in HK2 cell lines. The RT-qPCR (Fig. 2B) and western blot (Fig. 2F) results indicated that the expression of *Capn1* was upregulated in *NPHP1* knockdown HK2 cells. Immunofluorescence staining revealed similar results (Fig. 2G). These results demonstrated increased expression of calpain1 in the NPH.

### An impaired autophagy-lysosomal pathway was detected in the NPH models

**Fig. 3:**
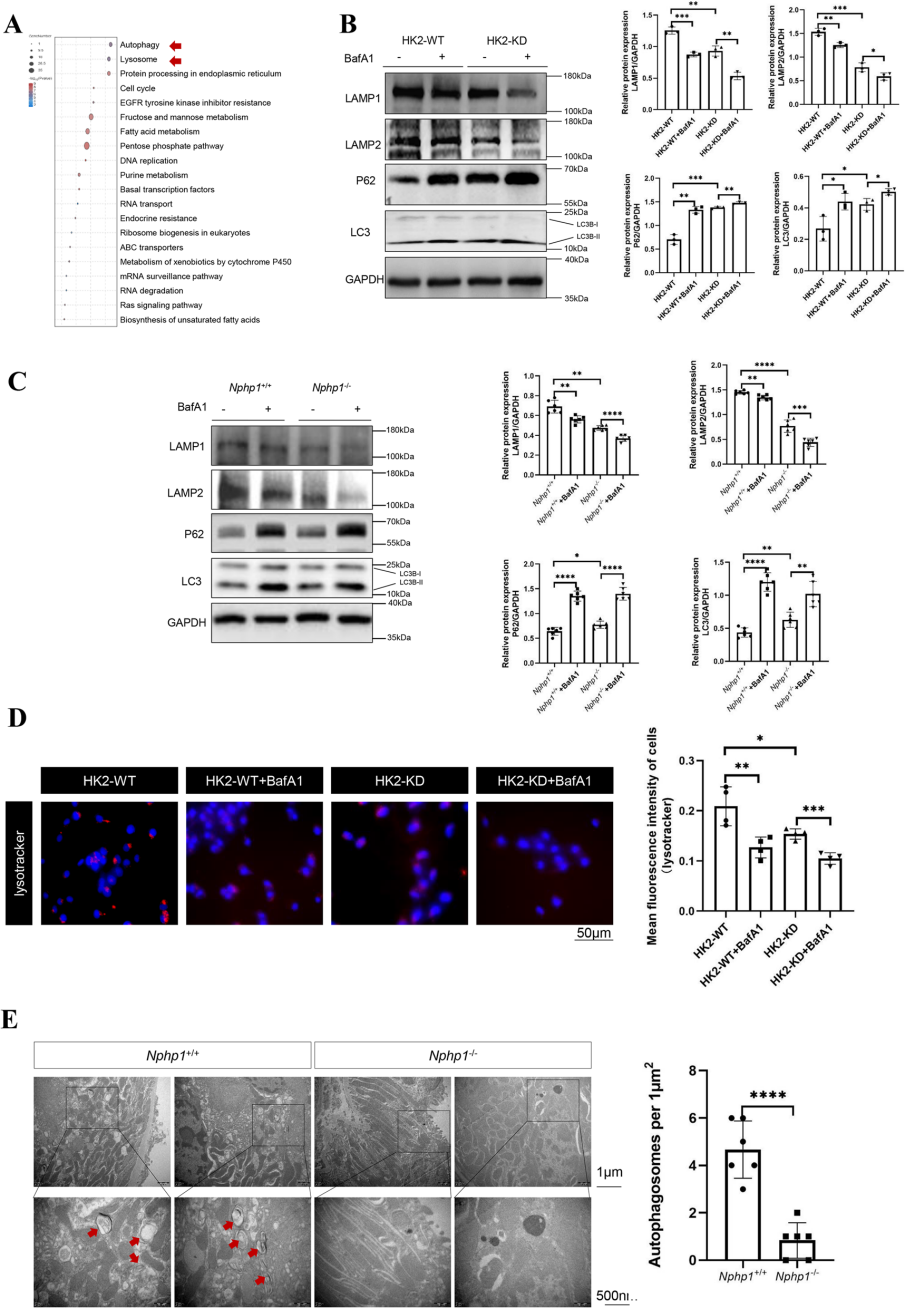
An impaired autophagy-lysosomal pathway was detected in the NPH models. **A** The top 20 enriched pathways in wild-type and *Nphp1* knockout mouse kidneys (*n* = 3). **B** Representative western blot analysis showing the expression of autophagic biomarkers and lysosomal biomarkers in wild-type and *NPHP1* knockdown HK2 cells. The result of the quantitative analysis was displayed as bar graphs. *n* = 3 in each group. **C** Representative western blot analysis showing the expression of autophagic biomarkers and lysosomal biomarkers in the kidneys of wild-type and *Nphp1* knockout mice. The result of the quantitative analysis was displayed as bar graphs. *n* = 6 in each group. **D** Wild-type and *NPHP1* knockdown HK2 cells were stained with LysoTracker™ Deep Red (red) and DAPI (blue). The results of the quantification of lysotracker staining in HK2 cells were evaluated. *n* = 4 in each group. Scale bar = 50 μm. **E** Transmission electron microscopy (TEM) analysis showing autophagosomes (arrow) in the kidneys of wild-type and *Nphp1* knockout mice. Scale bar = 1.0 µm (upper panel) or 500 nm (lower panel). The number of autophagosomes in mouse kidneys was quantified. *n* = 6 in each group. ^*^ P < 0.05; ^**^ P < 0.01; ^***^ P < 0.001; ^****^ P < 0.0001

Pathway enrichment analysis revealed that the autophagy and lysosome pathways were two of the top 20 pathways in NPH mouse kidney transcriptome (Fig. 3A, Table S3). Alterations in autophagic activity in NPH have not yet been reported. Since disorganized autophagy plays a role in renal ciliopathies Nowak and Edelstein 2020 and calpain1 can function as a regulator of autophagy activity Peintner et al. 2021, the autophagy process might also be involved in the mechanisms of NPH. Hence, autophagy was investigated in*NPHP1*-targeted HK2 cells and in mice. LC3B is a mammalian homolog of the yeast autophagy-related gene (Atg) 8. Upon autophagic activation, native LC3B (LC3B-I) is lipidated to form LC3B-II. LC3B-II is considered a marker of autophagosomes Weidberg et al. 2011. Western blot analysis revealed that endogenous LC3B-II levels were markedly elevated in*NPHP1* knockdown HK2 cells (Fig. 3B) and in *Nphp1* knockout mouse kidneys (Fig. 3C). Increases in LC3B-II levels can be caused by either an increase in autophagosome formation or a decrease in lysosome-mediated removal. To further elucidate the potential cause in NPH models, the distributions of the lysosomal markers LAMP1 and LAMP2 were studied. Compared with the respective controls, both the in vitro and in vivo models presented significantly decreased levels of LAMP1 and LAMP2 proteins (Fig. 3B, C). These results indicated impaired autophagic flux in NPH. For further verification, LysoTracker™ Deep Red, a dye that accumulates in acidic vesicles, was used to label autolysosomes. In our study, the LysoTracker signal was weaker in *NPHP1* knockdown HK2 cells than in control HK2 cells (Fig. 3D). To clearly visualize autophagic engulfment and degradation, transmission electron microscopy (TEM) was used. The number of autophagosomes was significantly decreased in the kidneys extracted from *Nphp1* knockout mice (Fig. 3E).

Autophagic flux, which is measured by comparing the prevalence of autophagosomes in the presence and absence of lysosomal inhibition, was recently introduced and is considered a better indicator of autophagic activity. We monitored body weight from the interference day (8w) to sacrificing day (24w) in male (Fig. S4 A) and female (Fig. S4B) mice with or without lysosomal inhibition by Bafilomycin A1. No significant body weight loss or gain was detected. Further accumulation of LC3B-II in *NPHP1*-targeted HK2 cells (Fig. 3B) and mouse kidneys (Fig. 3C) upon lysosomal inhibition by Bafilomycin A1 was observed. Taken together, these results indicate that the autophagy-lysosomal pathway is impaired in NPH models.

### An impaired autophagy-lysosomal pathway in NPH models induces tubulointerstitial fibrosis

**Fig. 4:**
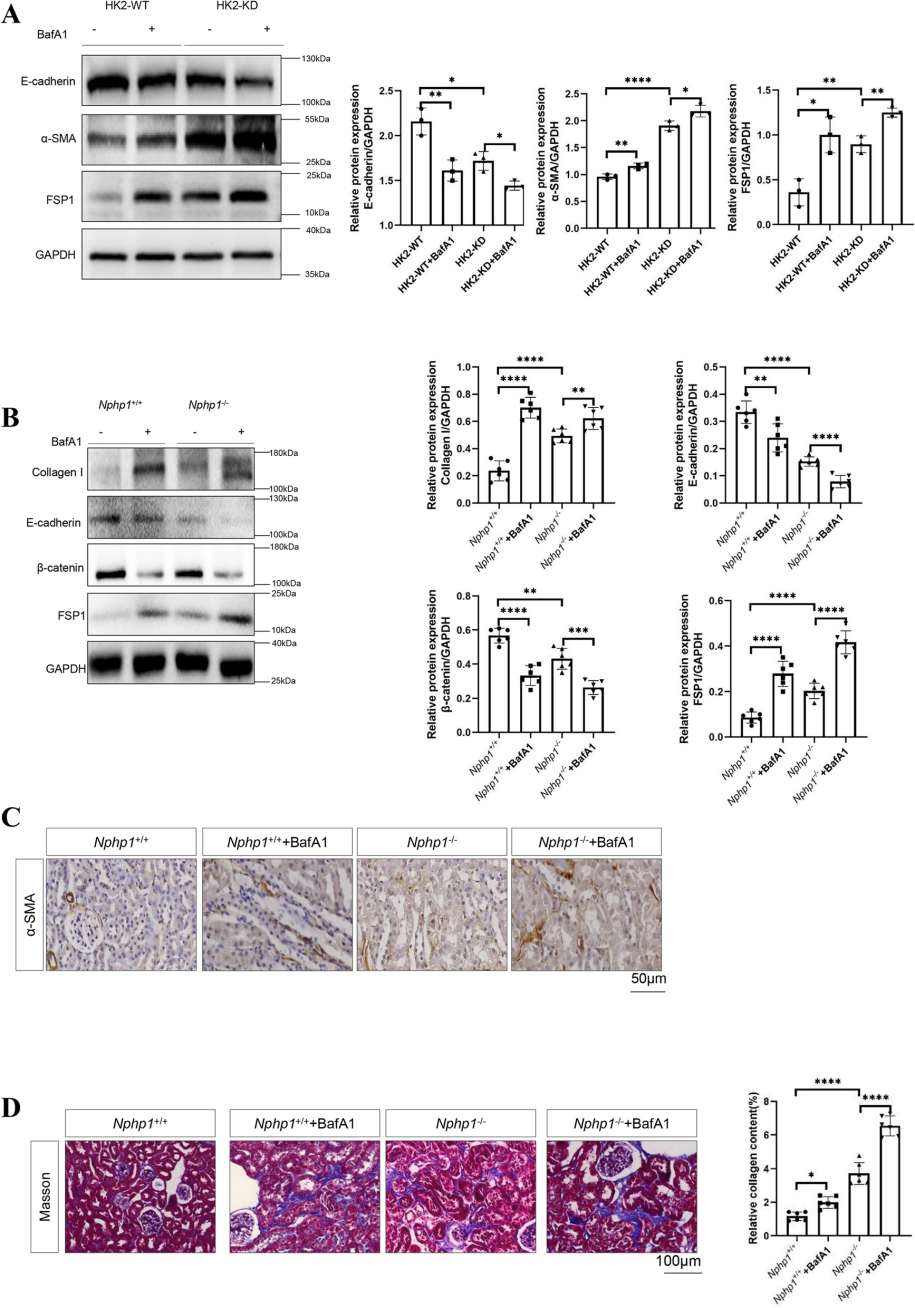
An impaired autophagy-lysosomal pathway in NPH models induces tubulointerstitial fibrosis. **A** Western blot analysis of the expression of epithelial and mesenchymal markers in wild-type and *NPHP1* knockdown HK2 cells. The result of the quantitative analysis was displayed as bar graphs. *n* = 3 in each group. **B** Western blot analysis of the expression of epithelial and mesenchymal markers in wild-type and *Nphp1* knockout mouse kidneys. The presence of two Collagen I bands (130–140 kDa) in the fourth lane can be attributed to the presence of multiple post-translational isoforms (e.g., glycosylated or acylated adducts) or different building units being synthesized (e.g., α1 or α2 chain) that were not resolved by electrophoresis. The result of the quantitative analysis was displayed as bar graphs. *n* = 6 in each group. **C** The expression of α-SMA in renal sections extracted from wild-type and *Nphp1* knockout mice was detected via immunohistochemical staining. Scale bar = 50 μm. **D** Masson’s trichrome (Masson) staining of renal sections was used to assess tubulointerstitial fibrosis in wild-type and *Nphp1* knockout mouse kidneys. Scale bar = 100 μm. The result of the quantitative analysis was displayed as bar graphs. *n* = 6 in each group. ^*^ *P* < 0.05; ^**^ P < 0.01; ^***^ P < 0.001; ^****^ P < 0.0001

Renal tubulointerstitial fibrosis represents a typical phenotype of NPH and promotes kidney dysfunction. Thus, figuring out the molecular basis of tubulointerstitial fibrosis might be conducive to understanding the mechanisms of NPH. To investigate whether autophagy-lysosomal pathway impairment influences interstitial fibrosis in NPH, the levels of several epithelial and mesenchymal markers were measured in *NPHP1*-targeted HK2 cells and mouse kidneys. *NPHP1* knockdown HK2 cells generally presented reduced expression of epithelial-specific genes (i.e., E-cadherin). On the other hand, increased levels of several mesenchymal-specific genes (i.e., α-SMA and FSP1) were observed (Fig. 4A). In whole kidneys extracted from *Nphp1* knockout mice, the levels of Collagen I, FSP1 (Fig. 4B) and α-SMA (Fig. 4C) were also increased, whereas the levels of E-cadherin and β-Catenin (Fig. 4B) were decreased.

To investigate the possible link between the autophagy-lysosomal pathway and tubulointerstitial fibrosis in NPH, the levels of epithelial and mesenchymal markers were measured in *NPHP1*-targeted HK2 cells and mouse kidneys subjected to lysosomal inhibition by Bafilomycin A1. Lysosomal inhibition resulted in a more severe reduction in E-cadherin expression and a greater increase in FSP1 and α-SMA (Fig. 4A) in *NPHP1* knockdown HK2 cells. In *Nphp1* knockout mice, a greater reduction of E-cadherin and β-Catenin expression (Fig. 4B) and greater increases of FSP1, Collagen I (Fig. 4B) and α-SMA (Fig. 4C) expression were observed. Masson staining also revealed a more severe phenotype of tubulointerstitial fibrosis with Bafilomycin A1 treatment in *Nphp1* knockout mice (Fig. 4D). Collectively, these results indicate that the impaired autophagic flux in NPH models induces tubulointerstitial fibrosis.

### Calpain1 inhibition improves the autophagy-lysosomal pathway in NPH models

**Fig. 5:**
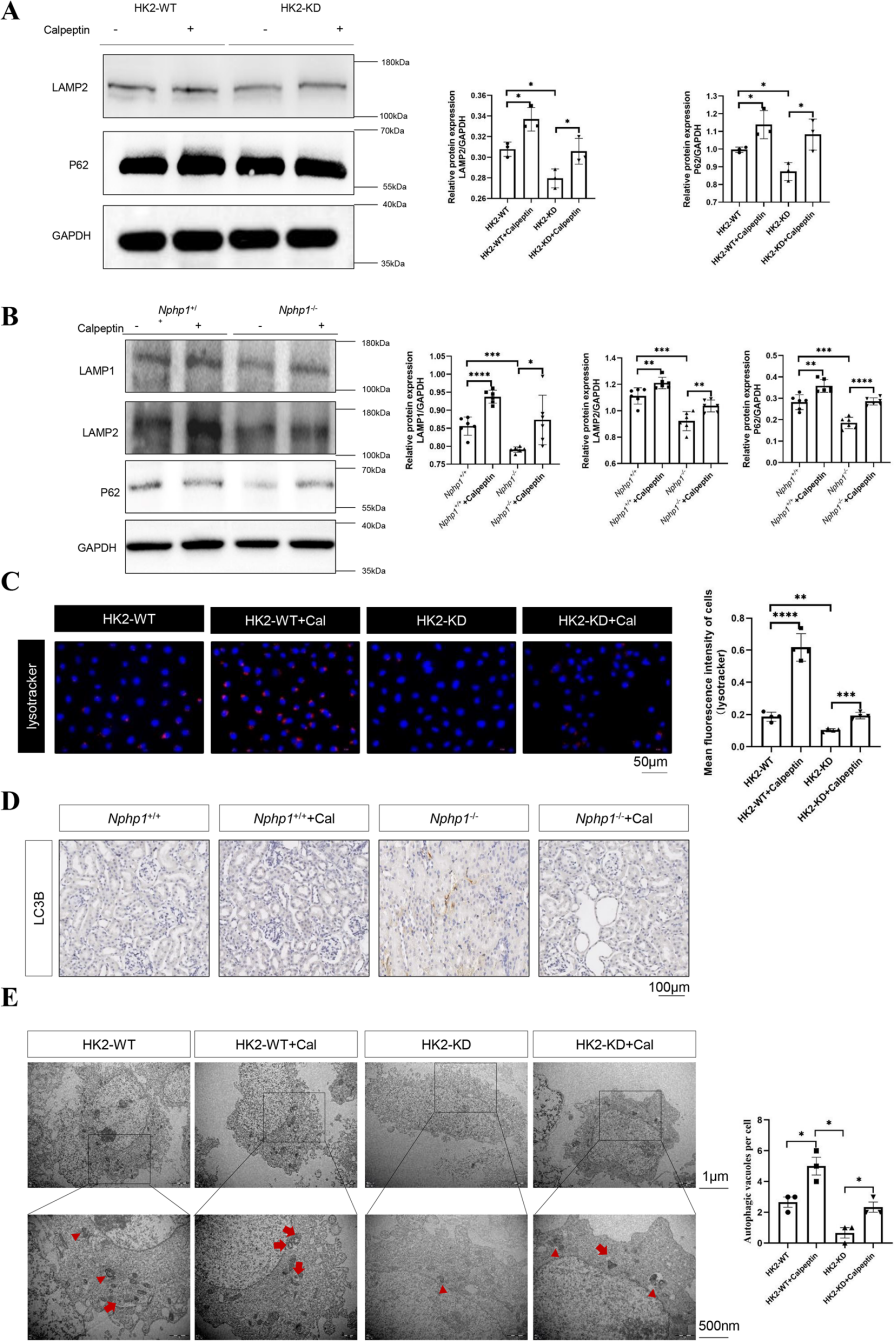
Calpain1 inhibition improves the autophagy-lysosomal pathway in NPH models. **A** HK2 cells were treated with or without calpeptin. Western blot was used to detect the expression of autophagic biomarkers and lysosomal biomarkers. The result of the quantitative analysis was displayed as bar graphs. *n* = 3 in each group. **B** Wild-type and *Nphp1* knockout mice were treated with or without calpeptin. Western blot was used to detect the expression of autophagic biomarkers and lysosomal biomarkers. The result of the quantitative analysis was displayed as bar graphs. *n* = 6 in each group. **C** LysoTracker™ Deep Red was used to label and track lysosomes in HK2 cells with or without calpeptin treatment. Quantitative analysis of lysosome signals in HK2 cells was performed. *n* = 4 in each group. Scale bar = 50 μm. **D** Immunohistochemical staining was used to detect the expression of LC3B in mouse kidneys with or without calpeptin treatment. Scale bar = 100 μm. **E** TEM analysis showing autophagosomes (red arrows) and autolysosomes (red triangles) in the wild-type and *NPHP1* knockdown HK2 cells with or without calpeptin treatment. Scale bar = 1.0 µm (upper panel) or 500 nm (lower panel). The number of autophagosomes and autolysosomes in HK2 cells was quantified. *n* = 3 in each group. ^*^ P < 0.05; ^**^ P < 0.01; ^***^ P < 0.001; ^****^ P < 0.0001

In light of the evidence that calpain1 induces lysosomal permeabilization by cleavage of LAMP2 Villalpando Rodriguez and Torriglia 2013, we investigated whether calpain1 upregulation in NPH correlated with impairment of the autophagy-lysosomal pathway. We monitored body weight from the interference day (8w) to the sacrificing day (24w) in male (Fig. S4 A) and female (Fig. S4B) mice treated with or without calpeptin, which is an inhibitor of calpain1. No significant body weight loss or gain was detected. P62 acts as an indicator of autophagy degradation, binding to ubiquitinated proteins to ensure their delivery to autophagosomes and degradation Liu et al. 2016. In the context of calpain1 inhibition by calpeptin, p62 accumulation was observed in NPH models (Fig.5A, B). In addition, a markedly stronger lysotracker signal was observed in *NPHP1* knockdown HK2 cells than in control HK2 cells (Fig. 5C). Furthermore, in both *NPHP1*-targeted HK2 cells and mouse kidneys, calpain1 inhibition by calpeptin increased the abundance of LAMP1 and LAMP2 (Fig. 5A, B). Calpain1 inhibition also reduced the LC3B level in the kidneys of NPH model mice (Fig. 5D). TEM images also revealed similar results, with increased numbers of autophagosomes and autolysosomes in *NPHP1* knockdown HK2 cells after calpeptin treatment (Fig. 5E). Overall, these data indicated that calpain1 inhibition improved the autophagy-lysosomal pathway in *NPNP1*-targeted HK2 cells and mouse kidneys.

### Calpain1 inhibition ameliorates tubulointerstitial fibrosis in NPH models

**Fig. 6:**
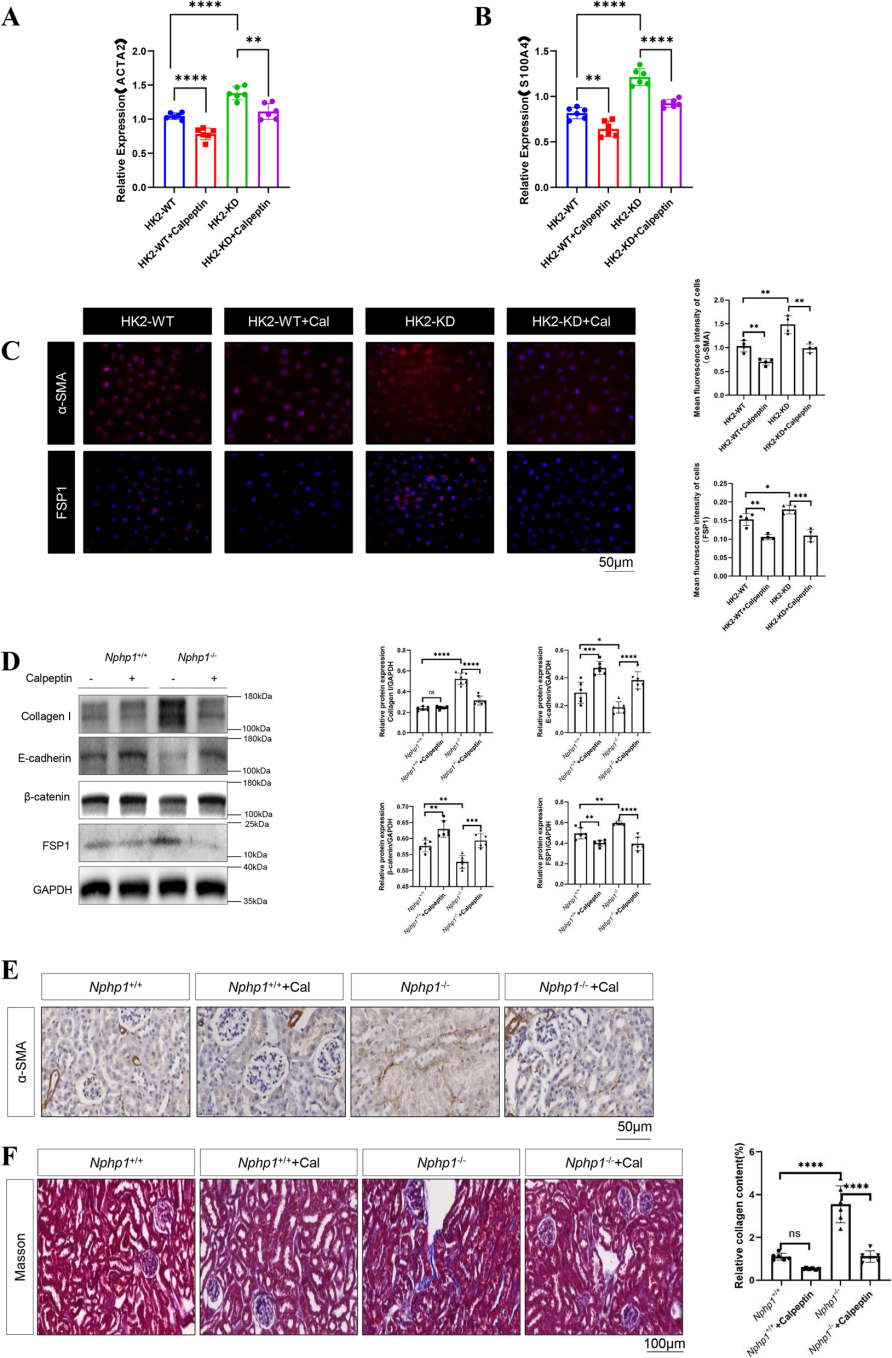
Calpain1 inhibition improves tubulointerstitial fibrosis in NPH models. **A** RT-qPCR analysis on expression of α-SMA in HK2 cells treated with or without calpeptin. β-actin was used as an internal control. *n* = 6 in each group. **B** RT-qPCR analysis on expression of FSP1 in HK2 cells treated with or without calpeptin. β-actin was used as an internal control. *n* = 6 in each group. **C** The expression of α-SMA and FSP1 in HK2 cells were detected by immunofluorescence. Scale bar = 50 μm. Quantitative analysis for α-SMA and FSP1 staining in HK2 cells with or without calpeptin treatment was evaluated. *n* = 4 in each group. **D** The expression of several epithelial and mesenchymal markers in mouse kidneys with or without calpeptin treatment was measured by western blot. The result of the quantitative analysis was displayed as bar graphs. *n* = 6 in each group. **E** The expression of α-SMA in renal sections was observed by immunohistochemistry. *n* = 6 in each group. Scale bar = 50 μm. **F** Masson staining of renal sections was used to assess the tubulointerstitial fibrosis. Scale bar = 100 μm. The result of the quantitative analysis was displayed as bar graphs. *n* = 6 in each group. ^*^ P < 0.05; ^**^ P < 0.01; ^***^ P < 0.001; ^****^ P < 0.0001

We investigated whether calpain1 inhibition resulted in antifibrotic effects on *NPHP1*-targeted HK2 cells and mouse kidneys. RT-qPCR analysis revealed decreased levels of mesenchymal markers, such as ACTA2 and S100 A4, after calpeptin treatment in *NPHP1* knockdown HK2 cells (Fig. 6A, B). Immunofluorescence staining also supported these findings (Fig. 6C). Compared with those of control mice, the kidneys of *Nphp1* knockout mice in the calpeptin group presented significantly higher β-catenin and E-cadherin levels and lower FSP1 and Collagen I levels (Fig. 6D). Immunohistochemical analysis of α-SMA (Fig. 6E) further confirmed that tubulointerstitial fibrosis was ameliorated by calpeptin treatment. Masson staining also revealed an improved phenotype of tubulointerstitial fibrosis with calpeptin treatment (Fig. 6F).

## Discussion

In this study, the expression of calpain1 was upregulated in both NPH patients and models. Mechanistically, calpain1 mediated autophagy-lysosomal pathway disorders and induced tubulointerstitial fibrosis. Here, when calpain1 was inhibited by calpeptin, the degree of autophagy-lysosomal disorder was decreased and the process of tubulointerstitial fibrosis was inhibited.

Calpains are capable of conducting selective protein hydrolysis to modulate substrate function. They are involved in the cell cycle maintenance, cellular motility, and signal transduction, as well as the regulation of cellular differentiation, migration, and diverse forms of programmed cell death. The calpain family is vital in activating or degrading proteins through signal-dependent pathway Nian and Ma 2021. Caspases play a vital role in apoptosis. Calpains can cleave caspase-3 and caspase-7 to activate apoptosis Gafni et al. 2009; Nelson et al. 2012. Caspase processing by caspase-12 via calpains contributes to the endoplasmic reticulum (ER) stress-induced cell death pathway Martinez et al. 2010. The critical event of mitochondria-mediated necrosis is the opening of the mitochondrial permeability transition pore (mPTP). Calpains induce Ca^2+^-dependent mPTP opening through the cleavage of complex I subunits on the electron transport chain Bonora et al. 2020; Arrington et al. 2006. The destruction of complex 1 causes mPTP sensitization. Furthermore, mitochondrial respiratory chain complex I Thompson et al. 2016; Shintani-Ishida and Yoshida 2015 and ATP synthases, such as ATP synthase subunit α (ATP5 A1) can be impaired by increased calpain activity in mitochondria, causing mitochondrial dysfunction and increased reactive oxygen species (ROS) production Ni et al. 2015; Ni et al. 2016.

The calpain family is involved in autophagy. The calpain family can lyse Beclin-1 to reduce autophagy activity Russo et al. 2011. Autophagy protein 5 (ATG5) participates in the formation of autophagosomes, which encase proteins or organelles, and serves as a substrate for calpain-mediated cleavage. Once cleaved by calpains, ATG5 not only prevents the formation of autophagosomes but also relocates to the mitochondria and interacts with B-cell lymphoma-extra large (Bcl-xL), leading to the release of cytochrome C Yousefi et al. 2006. Calpains can also cleave LAMP2 on the lysosomal membrane, resulting in lysosome-dependent cell death instead of autophagy Villalpando Rodriguez and Torriglia 2013; Arnandis et al. 2012. In our study, a link between elevated calpain1 expression and impaired autophagic flux was discovered, and calpain1 inhibition improved autophagy disorders in NPH. The regulatory role of calpain1 in autophagy is likely mediated by its proteolytic activity, with lysosomal membrane proteins emerging as putative substrates. Although current imaging data lack definitive evidence for calpain1's spatial colocalization with specific subcellular compartments, this knowledge gap could be systematically resolved by employing advanced techniques such as live-cell imaging coupled with super-resolution microscopy in subsequent investigations.

Previous studies have indicated that renal tubulointerstitial fibrosis and the autophagy-lysosomal pathway are interconnected in the context of chronic kidney diseases Hewitson 2009; Ding and Choi 2014. Autophagy-lysosomal pathway impairment was observed in NPH models in our study. When the autophagy-lysosomal pathway was further inhibited by Bafilomycin A1, renal tubulointerstitial fibrosis became more prominent. This result revealed that autophagy-lysosomal pathway impairment at least partly induced tubulointerstitial fibrosis in NPH models.

An impaired autophagy-lysosomal pathway might induce the development of renal tubulointerstitial fibrosis through various pathways. Defects in the autophagy-mediated turnover of fibronectin have been linked to excessive deposition of fibronectin in the extracellular matrix, leading to renal fibrosis Xu et al. 2016. Activation of the NOD-like receptor protein 3 (NLRP3) inflammasome can lead to the production of proinflammatory cytokines, which contribute to renal inflammation and fibrosis Wang et al. 2020. Autophagy has been shown to attenuate tubulointerstitial fibrosis by modulating the NLRP3 inflammasome signaling pathway Wu et al. 2021. Enhancing autophagy can lead to the degradation of NLRP3, thereby reducing inflammation and fibrosis in the kidney Guo et al. 2017. Conversely, impaired autophagy has been linked to increased inflammation and interstitial fibrosis in chronic kidney diseases Zhang and Wang 2019. Furthermore, epithelial-mesenchymal transition (EMT) has been identified as a significant event in the progression of renal tubulointerstitial fibrosis Slattery et al. 2006. Impaired autophagic flux has been found to play a critical role in renal tubulointerstitial fibrosis by promoting EMT Liang et al. 2022.

In our study, NPH models presented generally reduced expression of epithelial-specific genes (i.e. E-cadherin), and increased expression of several mesenchymal-specific genes (i.e. α-SMA and FSP1). Lysosomal inhibition by Bafilomycin A1 resulted in a more severe reduction in E-cadherin expression and a greater increase in FSP1 and α-SMA. These results indicate that impaired autophagy-lysosomal pathway activity might induce tubulointerstitial fibrosis by promoting EMT in NPH.

Whether the autophagy-lysosomal pathway is the only pathological process through which calpain1 induces tubulointerstitial fibrosis in NPH remains unknown. Another unresolved question in our study is the upstream mechanism of calpain1 elevation in NPH.

## Conclusions

**Fig. 7:**
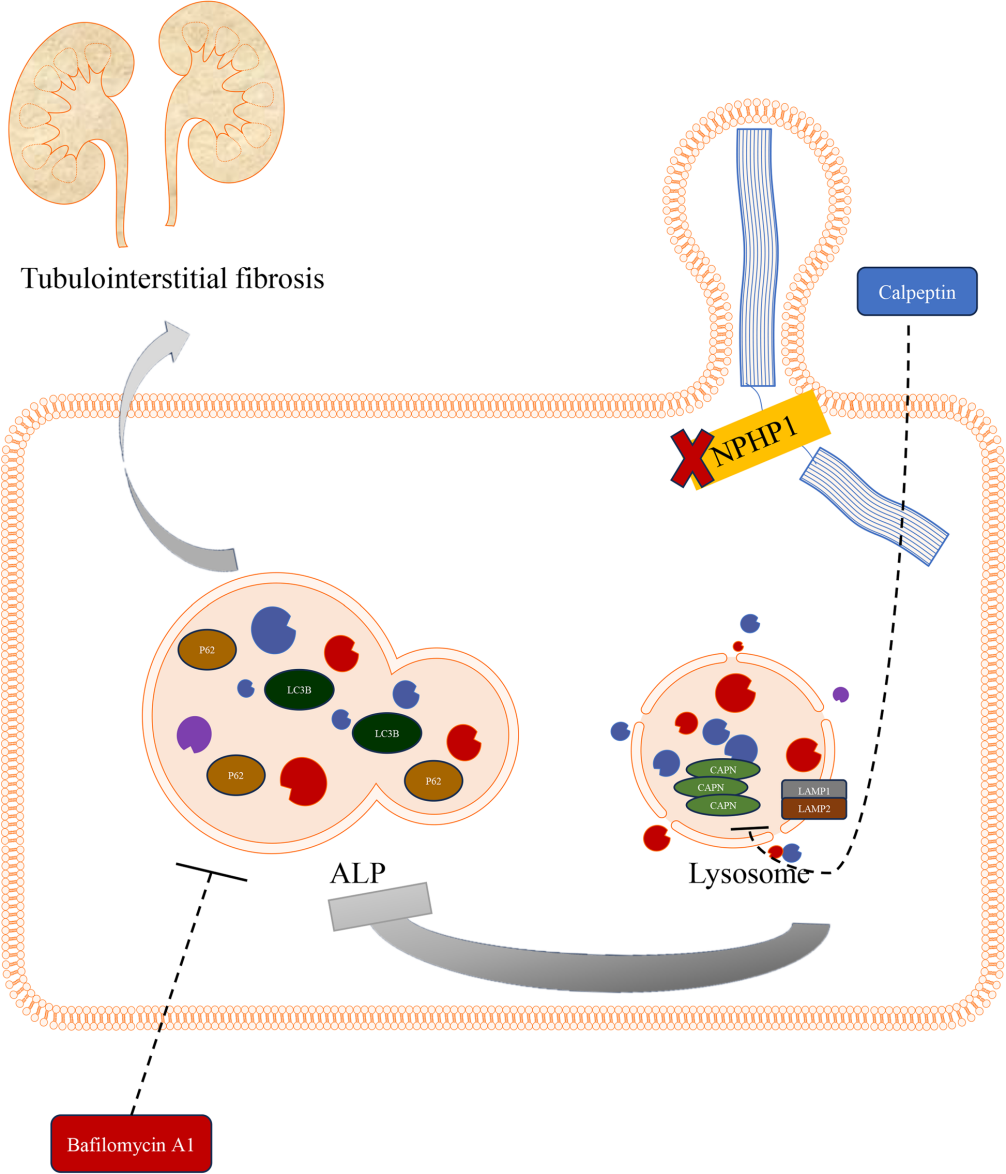
Graphical abstract. In the context of NPH (*NPHP1*-deficiency), upregulation of calpain1(CAPN) and impairment of autophagy-lysosomal pathway were detected. The impaired autophagy-lysosomal pathway might be caused by the upregulation of calpain1 and could induce tubulointerstitial fibrosis. When calpain1 was inhibited by calpeptin, the autophagy-lysosomal pathway was enhanced. Furthermore, calpain1 inhibition resulted in antifibrotic effects in NPH. ALP, autophagy-lysosomal pathway

We demonstrated the upregulation of calpain1 and impairment of the autophagy-lysosomal pathway in NPH. An impaired autophagy-lysosomal pathway can induce tubulointerstitial fibrosis in NPH. When calpain1 was inhibited, the autophagy-lysosomal pathway was improved. Furthermore, calpain1 inhibition resulted in antifibrotic effects in NPH (Fig. 7). Together, our results support calpain1 as a potential diagnostic and therapeutic target for NPH.

## Supplementary Information

Additional file 1.

Additional file 2.

Additional file 3.

Additional file 4.

Additional file 5.

Additional file 6.

Additional file 7.

## Data Availability

No datasets were generated or analysed during the current study.

## Abbreviations

NPH: Nephronophthisis
PKD: Polycystic kidney disease
I/R: Ischemia/reperfusion
CKD: Chronic kidney disease
ADPKD: Autosomal dominant polycystic kidney disease
SPF: Specific pathogen-free
IHC: Immunohistochemistry
BSA: Bovine serum albumin
DEGs: Differentially expressed genes
HPA: Human protein atlas
Atg: Autophagy-related gene
LAMP: Lysosome associated membrane protein
TEM: Transmission electron microscopy
GAPDH: Glyceraldehyde 3-phosphate dehydrogenase
LC3: Light chain 3
P62: Sequestosome 1
α-SMA: Alpha smooth muscle actin
FSP1: Fibroblast specific protein 1
mPTP: Mitochondrial permeability transition pore
ROS: Reactive oxygen species
UUO: Unilateral ureteric obstruction
TGFβ1: Transforming growth factor β1
TECs: Tubular epithelial cells
