# Supplementary figures and images for: Calpain1 inhibition enhances autophagy-lysosomal pathway and ameliorates tubulointerstitial fibrosis in Nephronophthisis
Source: Mol Med. 2025 May 3;31:166. doi: 10.1186/s10020-025-01231-4 (PMC12049798; doi:10.1186/s10020-025-01231-4)

Figure S1

A

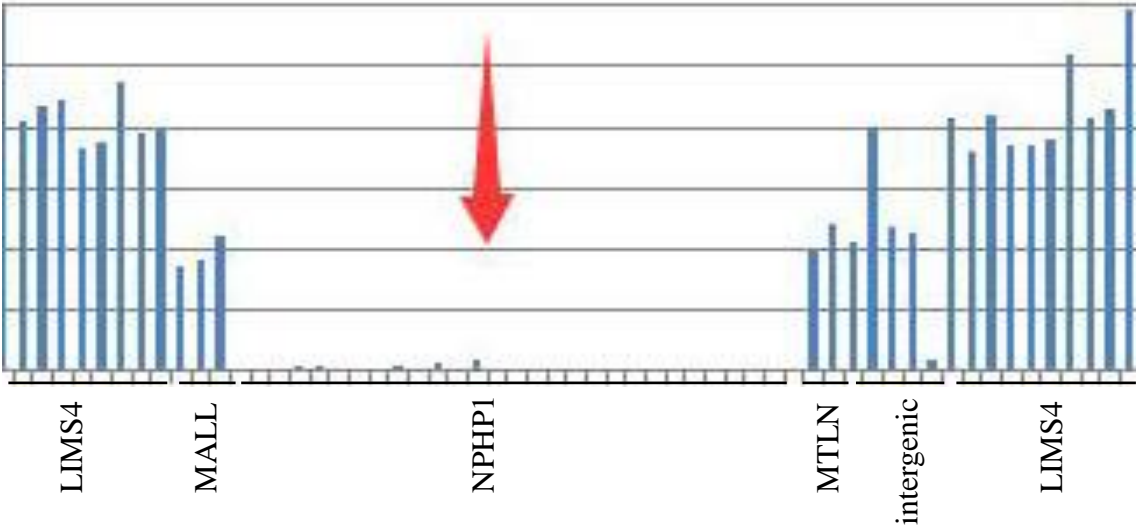

B

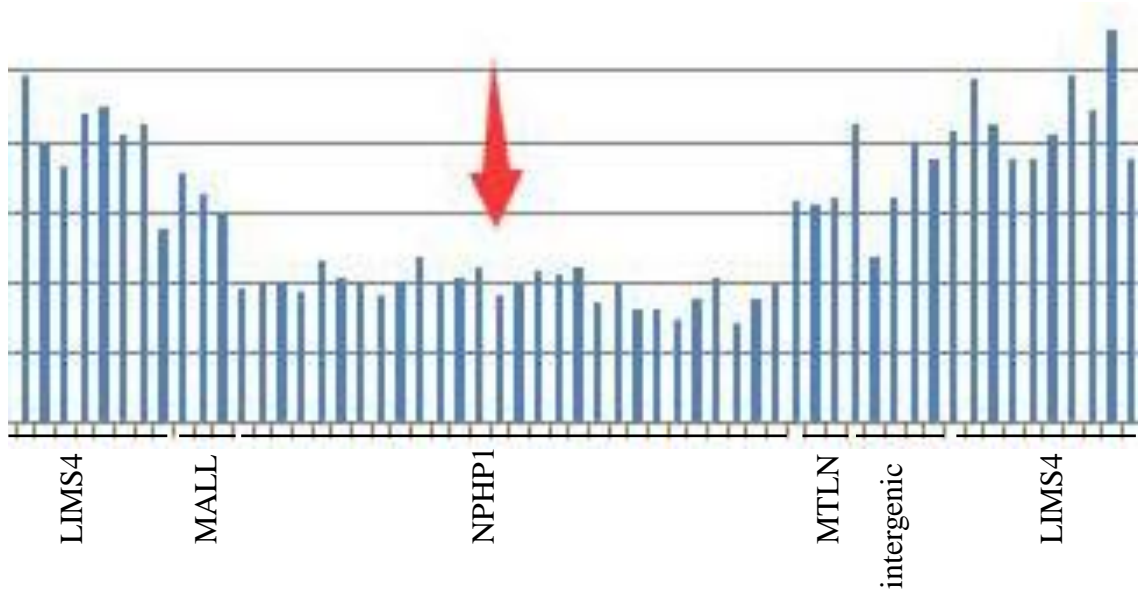

C

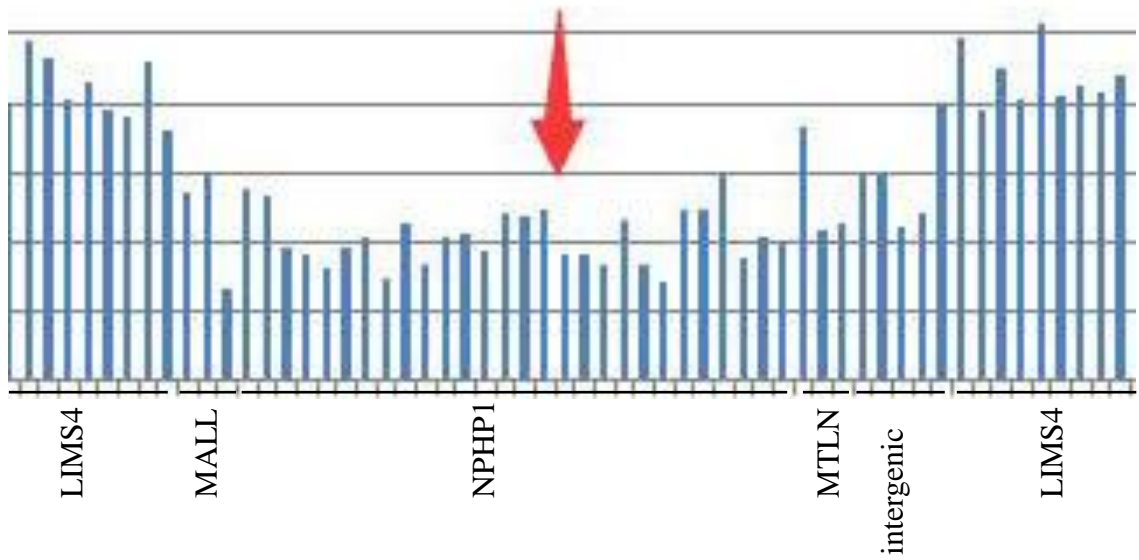

Supplement: Supplementary file 1 — Additional file 1. [file 10020_2025_1231_MOESM1_ESM.pdf]

A

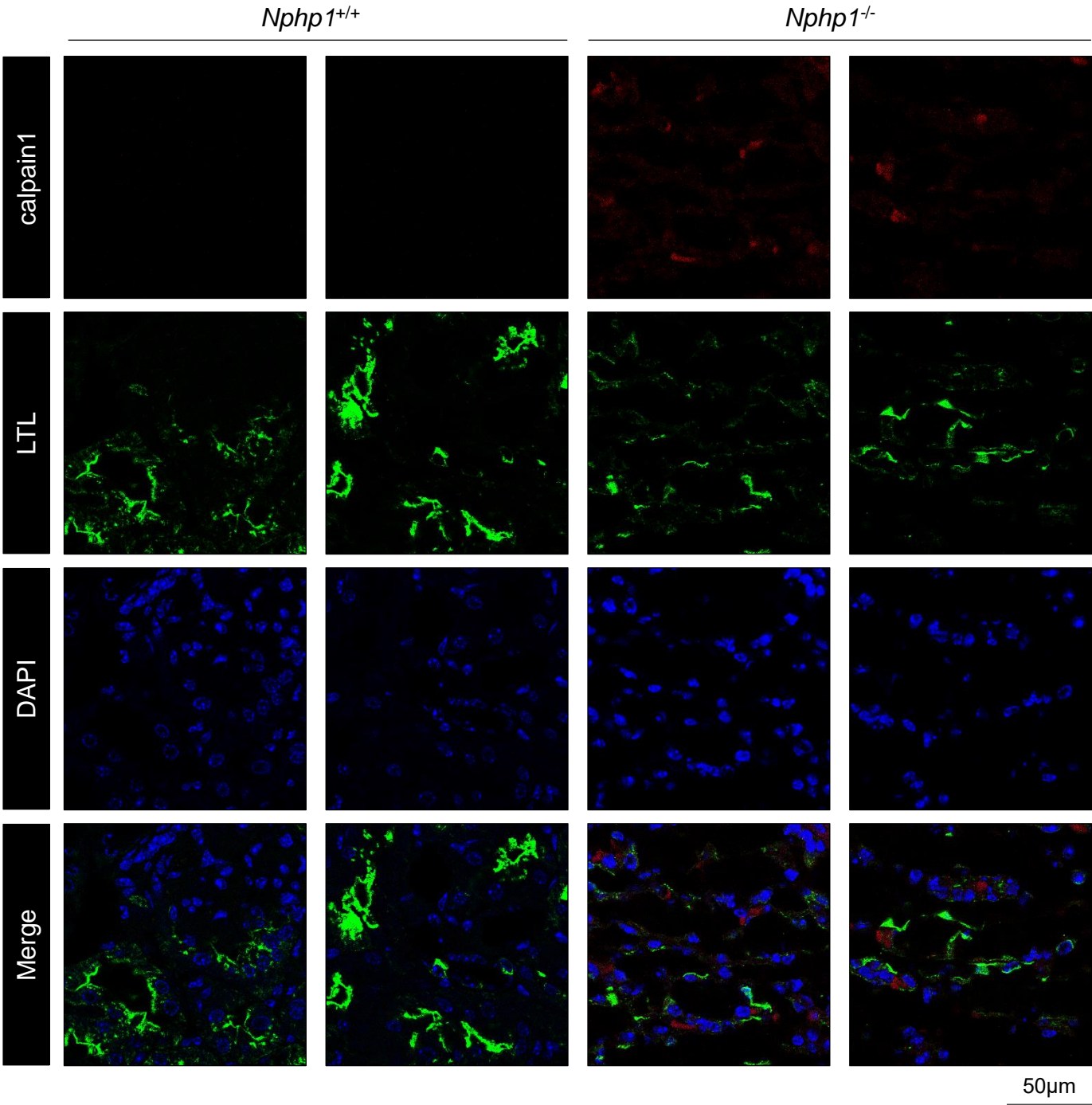

Supplement: Supplementary file 2 — Additional file 2. [file 10020_2025_1231_MOESM2_ESM.pdf]

A

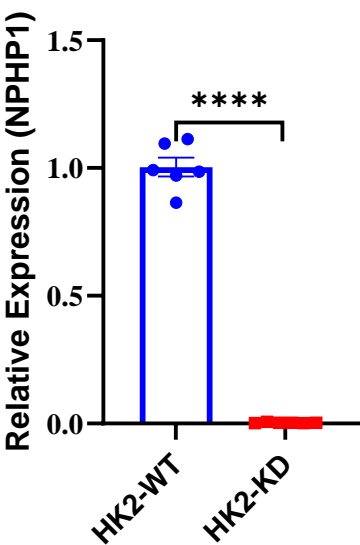

B

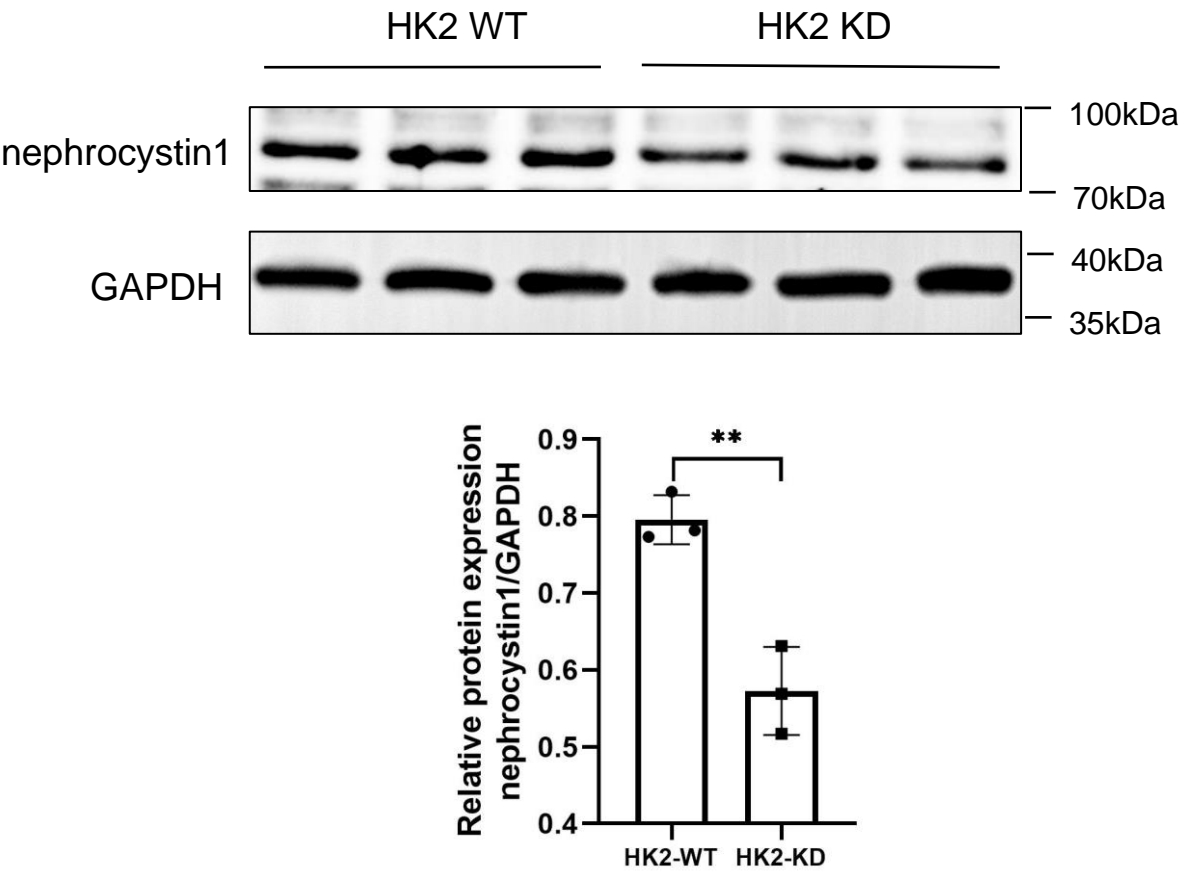

Supplement: Supplementary file 3 — Additional file 3. [file 10020_2025_1231_MOESM3_ESM.pdf]

Figure S4

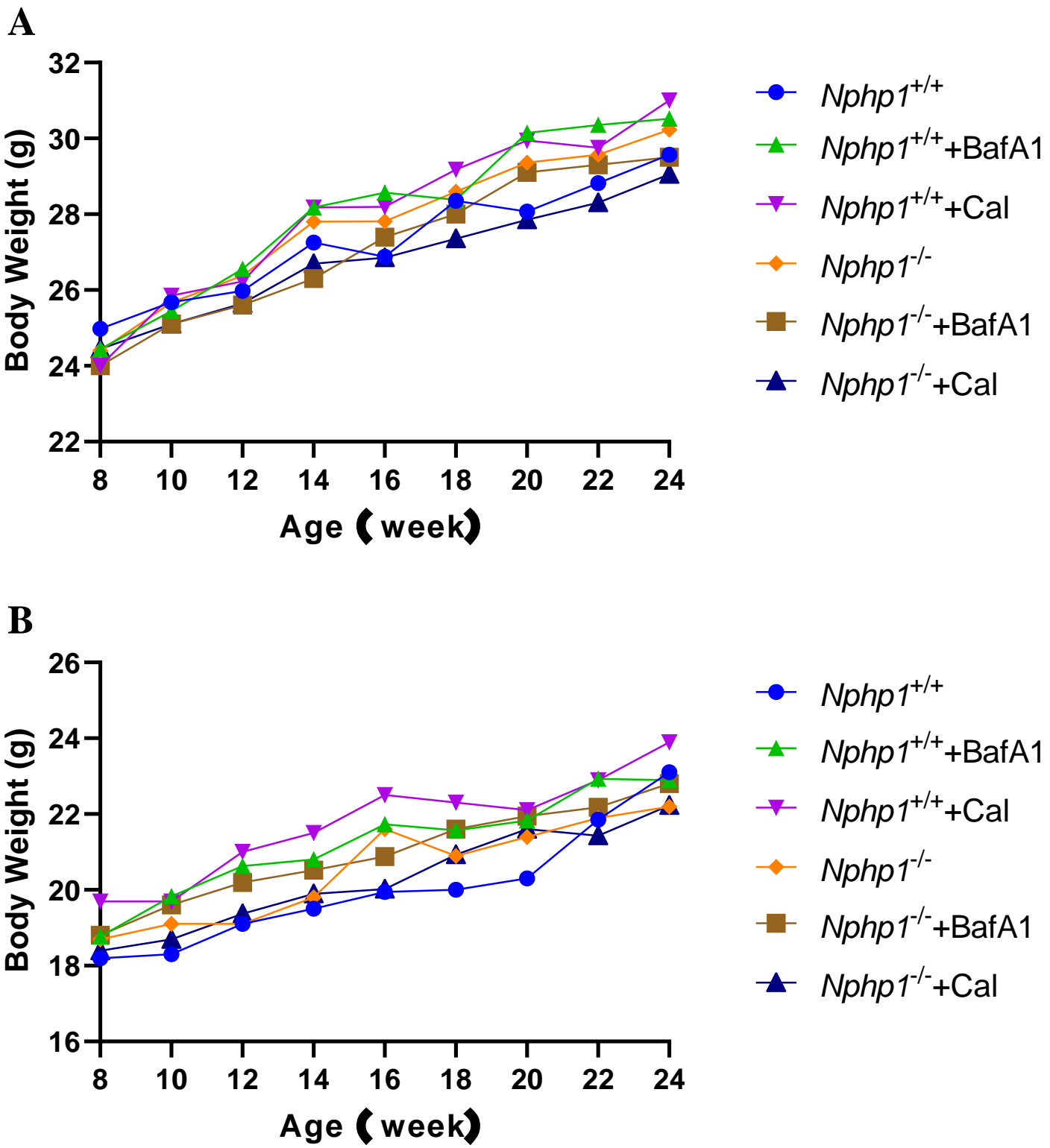

Supplement: Supplementary file 4 — Additional file 4. [file 10020_2025_1231_MOESM4_ESM.pdf]
